# Association between high-sensitivity cardiac troponin I measured at emergency department and complications of emergency coronary artery bypass grafting

**DOI:** 10.1038/s41598-019-53047-y

**Published:** 2019-11-15

**Authors:** Jungchan Park, Seung-Hwa Lee, Jeong Jin Min, Jong-Hwan Lee, Ji Hye Kwon, Ja Eun Lee, Jin-Ho Choi, Young Tak Lee, Wook Sung Kim, Myungsoo Park, Ji Su Jang, Sangmin Maria Lee

**Affiliations:** 1 Department of Anesthesiology and Pain Medicine, Samsung Medical Center, Sungkyunkwan University School of Medicine, Seoul, Korea; 2 Division of Cardiology, Department of Medicine, Heart Vascular Stroke Institute, Samsung Medical Center, Sungkyunkwan University School of Medicine, Seoul, Korea; 3 Department of Emergency Medicine, Samsung Medical Center, Sungkyunkwan University School of Medicine, Seoul, Korea; 4 Department of Thoracic and Cardiovascular Surgery, Samsung Medical Center, Sungkyunkwan University School of Medicine, Seoul, Korea; 5 Department of Internal Medicine, Hallym University Dongtan Sacred Heart Hospital, Hwaseong, Korea; 6 Department of Anesthesiology and Pain Medicine, College of Medicine, Kangwon National University, Gangwondaehak-gil, Chuncheon-si, Gangwon-do Republic of Korea

**Keywords:** Predictive markers, Predictive markers, Predictive markers, Interventional cardiology, Interventional cardiology, Interventional cardiology

## Abstract

High-sensitivity cardiac troponin I (hs-cTnI) is a widely used biomarker to identify ischemic chest pain in the Emergency Department (ED), but the clinical impact on emergency coronary artery bypass grafting (eCABG) remains undetermined. We aimed to evaluate the clinical impact of hs-cTnI measured at the ED by comparing outcomes of eCABG in patients with non–ST-segment–elevation acute coronary syndrome (NSTE-ACS) which comprises unstable angina (UA) and non–ST-segment–elevation myocardial infarction (NSTEMI). From January 2012 to March 2016, 242 patients undergoing eCABG were grouped according to serum hs-cTnI level in the ED. The primary endpoint was major cardiovascular cerebral event (MACCE) defined as a composite of all-cause death, myocardial infarction, repeat revascularization, and stroke. The incidence of each MACCE composite, in addition to postoperative complications such as acute kidney injury, reoperation, atrial fibrillation, and hospital stay duration were also compared. Patients were divided into two groups: UA [<0.04 ng/mL, n = 102] and NSTEMI [≥0.04 ng/mL, n = 140]. The incidence of MACCE did not differ between the two groups. Postoperative acute kidney injury was more frequent in the NSTEMI group after adjusting for confounding factors (6.9% vs. 23.6%; odds ratio, 2.76; 95% confidence interval, 1.09–6.99; *p*-value = 0.032). In-hospital stay was also longer in the NSTEMI group (9.0 days vs. 15.4 days, *p*-value = 0.008). ECABG for UA and NSTEMI patients showed comparable outcomes, but hs-cTnI elevation at the ED may be associated with immediate postoperative complications.

## Introduction

Stratifying patients with ischemic cardiac symptoms in the Emergency Department (ED) is critical but challenging. Especially, the subgroups of non–ST-segment–elevation acute coronary syndrome (NSTE-ACS) which comprises unstable angina (UA), and non–ST-segment–elevation myocardial infarction (NSTEMI) present indistinguishable clinical and electrocardiographic features, but the clinical impact of emergency coronary artery bypass grafting (eCABG) on these subgroups remains uncertain^1^. Serial testing of cardiac biomarkers has shown excellent diagnostic performance, and the development of high-sensitivity cardiac troponin assay has led to reclassification of NSTE-ACS^2^. That is, the patients with resting chest pain who were originally thought to have UA could be reclassified as having an NSTEMI^1^. On previous studies, the clinical significance of high-sensitivity cardiac troponin was validated among acute coronary syndrome (ACS) patients in the ED setting, and the subgroups of NSTE-ACS, classified by high-sensitivity cardiac troponin, have also shown different clinical outcomes during medical or placebo therapy^3,4^. However, current guidelines lack details on surgical coronary revascularization^5–8^.

Although the overall outcome of coronary artery bypass grafting (CABG) has significantly improved over time with well-established benefits in multi-vessel disease with complex lesions^9,10^, eCABG for ACS patients represent challenging subgroup given their high risk characteristics^11^. The current guidelines suggest a limited role of eCABG in the acute phase of ST-segment–elevation myocardial infarction (STEMI)^5–8^. So, understanding the clinical impact of high-sensitivity cardiac troponin on eCABG may be helpful in determining treatment strategy for NSTE-ACS patients in the ED. Therefore, we aimed to compare the postoperative outcome of eCABG among the subgroups of NSTE-ACS, classified by high-sensitivity cardiac troponin I (hs-cTnI) measured at the ED.

## Results

**Figure 1:**
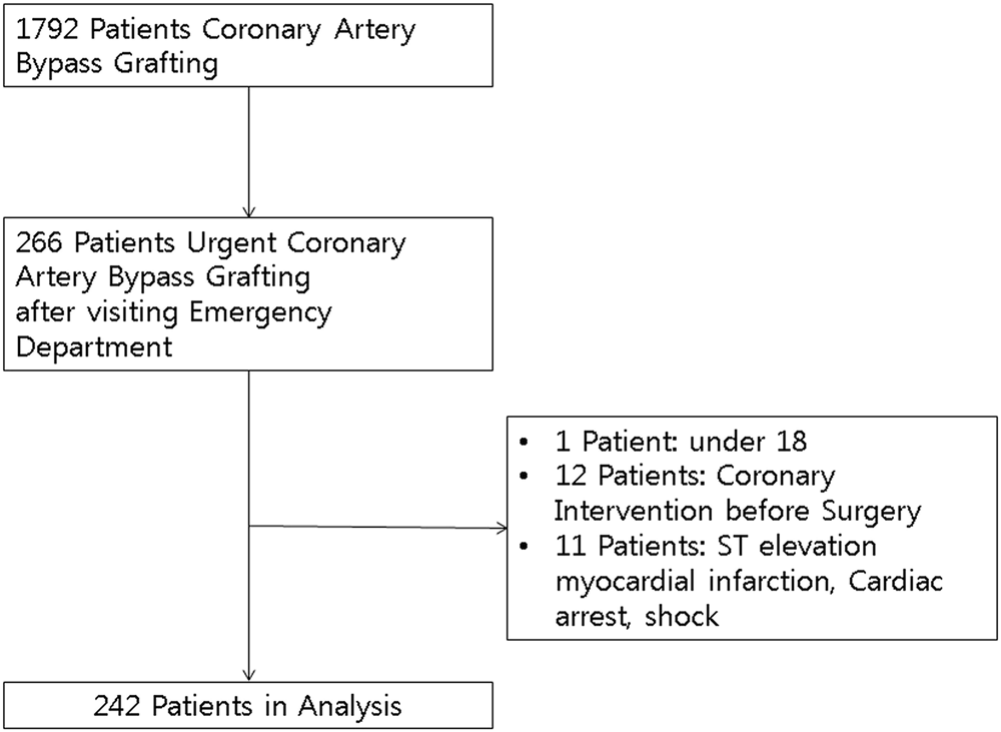
Patient flowchart.

Among the 1,792 patients who underwent CABG from January 2012 to March 2016, 266 patients underwent eCABG, and 242 patients met inclusion criteria for analysis. The flowchart of patients is shown in Fig. 1. Enrolled patients were divided into two groups, UA [<0.04 ng/mL, n = 102] and NSTEMI [≥0.04 ng/mL, n = 140], according to peak hs-cTnI level in the ED. Average peak hs-cTnI measured in the ED was 0.013 (±0.008) in the UA group and 9.887 (±30.4) in the NSTEMI group (*p*-value < 0.001).

### Baseline characteristics

Preoperative variables are presented in Table 1, and operative variables are shown in Table 2. Preoperative creatinine level was significantly higher in the NSTEMI group (0.98 [±0.29] mg/dL vs. 1.28 [±1.35] mg/dL; *p*-value < 0.001), but preoperatively measured ejection fraction and hemoglobin level were significantly lower in the NSTEMI group (56.2 [±11.1] % vs. 48.2 [±12.9] %; *p*-value = 0.016, 13.7 [±1.6] g/dL vs. 13.2 [±2.0] g/dL; *p*-value = 0.029, respectively) (Table 1). Among operative variables, completion of CABG with the off-pump technique was lower in the NSTEMI group (90.2% vs. 66.4%; *p*-value < 0.001), but the use of aortic modulation was higher (6.9% vs. 21.4%; *p*-value = 0.002) (Table 2). Other variables did not significantly differ among two groups.

**Table 1:** Preoperative variables.

	UA (N = 102)	NSTEMI (N = 140)	*p*-value
Age*	63.4 (±10.0)	63.1 (±10.8)	0.382
Male	81 (79.4)	106 (75.7)	0.498
Hypertension	65 (63.7)	94 (67.1)	0.58
Diabetes	47 (46.1)	64 (45.7)	0.955
COPD	2 (2.0)	1 (0.7)	0.575
Stroke	16 (15.7)	17 (12.1)	0.453
Peripheral vascular disease	8 (7.8)	15 (10.7)	0.512
Smoking	34 (33.3)	59 (42.1)	0.164
Chronic kidney disease	1 (1)	12 (8.6)	0.01
Creatinine, mg/dL	0.98 (±0.29)	1.28 (±1.35)	<0.001
Ejection fraction, %	56.2 (±11.1)	48.2 (±12.9)	0.016
Preoperative anemia	14 (13.7)	38 (27.1)	0.017
Hemoglobin, g/dL	13.7 (±1.6)	13.2 (±2.0)	0.029
**Prior revascularization**			
CABG	0	2 (1.4)	0.51
PCI	14 (13.7)	22 (15.7)	0.668
**Medication**			
Statin	44 (43.1)	51 (36.4)	0.291
Antiplatelet agent	82 (80.4)	106 (75.7)	0.388
**Diseased vessels**			
Single vessel disease	6 (5.9)	10 (7.1)	0.697
Double vessel disease	28 (27.5)	29 (20.7)	0.355
Triple vessel disease	68 (66.7)	101 (72.1)	0.396
Left main disease	28 (27.5)	37 (26.4)	0.859
**Laboratory tests**			
Hemoglobin (g/dl)*	13.7 (±1.6)	13.2 (±2.0)	0.061
Platelet (x10³/μl)*	205 (±51)	208 (±52)	0.623
INR*	1.00 (±0.08)	1.03 (±0.16)	0.094
Hs-cTnI (ng/ml)*	0.013 (±0.008)	9.887 (±30.4)	0.001

Values are n (%) or mean (±SD)*.

UA; unstable angina; NSTEMI; non–ST-segment–elevation myocardial infarction; PCI: Percutaneous coronary intervention; COPD: chronic obstructive pulmonary disease; hs-cTnI: high-sensitivity cardiac troponin I.

**Table 2:** Operative variables.

	UA (N = 102)	NSTEMI (N = 140)	*P*-value
Off-pump technique	92 (90.2)	93 (66.4)	<0.0001
Aortic modulation	7 (6.9)	30 (21.4)	0.002
**Graft**			
Total number	3.96 (±1.27)	4.10 (±1.40)	0.427
LITA	101 (99.0)	136 (97.1)	0.401
RITA	92 (90.2)	122 (87.1)	0.544
RGEA	4 (3.9)	8 (5.7)	0.766
Radial artery	0	0	
SVG	1 (1)	0	0.421

Values are n (%) or mean (±SD).

UA; unstable angina; NSTEMI; non–ST-segment–elevation myocardial infarction; LITA: left internal thoracic artery; RITA: right internal thoracic artery; RGEA: right gastroepiploic artery; SVG: saphenous vein graft.

### Clinical outcomes

**Figure 2:**
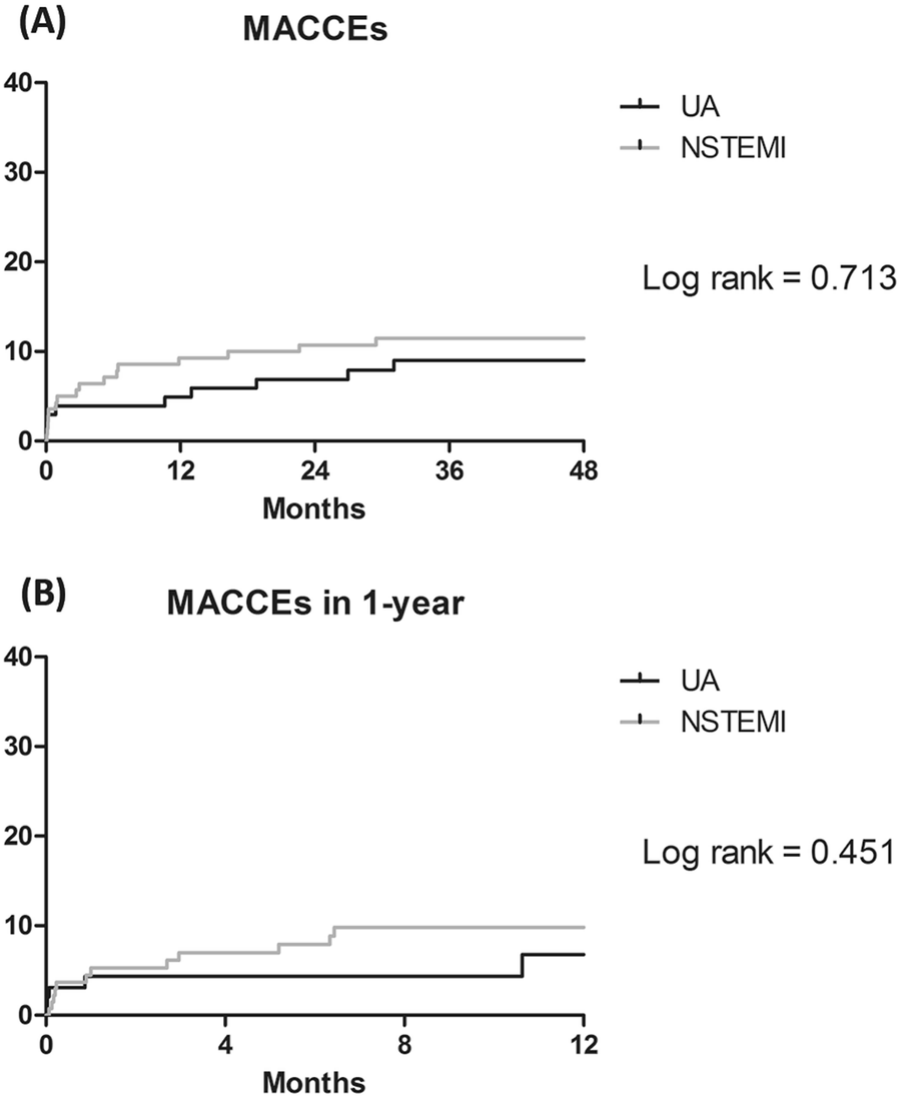
The Kaplan-Meier Curves for major adverse cardiovascular and cerebral events during (**A**) overall follow-up and (**B**) 12-month follow-up (x: duration of follw-up period, y: percentage of patients with major adverse cardiovascular and cerebral events).

Clinical outcomes are presented in Table 3. The median follow-up periods were 50.0 (32.8–61.5) months in the UA group and 56.7 (32.2–71.7) months in the NSTEMI group (*p*-value = 0.066). After adjustment, the incidence of MACCE during the overall follow-up period was not significantly different (8.8% vs. 11.4%; odds ratio [OR], 1.04; 95% confidence interval [CI], 0.43–2.51; *p-*value = 0.926). The incidence of MACCE during one year, 30-day, or in-hospital follow-ups did not differ between the two groups. Non-inferiority test showed that mean difference of MACCE between UA and NSTEMI was –2.6% (95% CI −10.2% to 5.01%). At 5.2% of non-inferiority margin, p-value was 0.022. Survival curves for MACCE are presented in Fig. 2.

**Table 3:** Clinical outcomes.

	UA (N = 102)	NSTEMI (N = 140)	Unadjusted HR/OR (CI 95%)	*P*-value	Adjusted HR/OR (CI 95%)	*P*-value
MACCE within follow-up period*	9 (8.8)	16 (11.4)	1.29 (0.57–2.92)	0.543	1.04 (0.43–2.51)	0.926
All-cause death*	2 (2.0)	5 (3.6)	1.86 (0.36–9.61)	0.457	1.30 (0.20–8.37)	0.78
Repeat revascularization*	4 (3.9)	8 (5.7)	1.43 (0.43–4.76)	0.560	1.56 (0.42–5.74)	0.504
Myocardial infarction*	2 (2.0)	0				
Stroke*	2 (2.0)	4 (2.9)	1.35 (0.25–7.39)	0.733	0.97 (0.15–6.50)	0.97
MACCE within 1-year follow-up*	5 (4.9)	12 (8.6)	1.49 (0.52–4.24)	0.454	1.46 (0.47–4.50)	0.513
MACCE within 30-days follow-up*	4 (3.9)	7 (5.0)	1.19 (0.35–4.07)	0.781	0.81 (0.20–3.28)	0.769
MACCE during in-hospital stay	3 (2.9)	9 (6.4)	2.27 (0.60–8.59)	0.229	1.75 (0.39–7.89)	0.465
Postoperative acute kidney injury	7 (6.9)	33 (23.6)	4.19 (1.77–9.90)	0.001	2.76 (1.09–6.99)	0.032
Stage 2	0	7 (5)				
Stage 3	0	2 (1.4)				
Atrial fibrillation	16 (15.7)	37 (26.4)	1.93 (1.01–3.71)	0.048	1.72 (0.83–3.57)	0.145
Reoperation	1 (1.0)	12 (8.6)	9.47 (1.21–74.04)	0.032	5.13 (0.60–44.1)	0.137
Bleeding	0	5 (3.6)				
Others	1 (1.0)	8 (5.7)	6.12 (0.75–49.74)	0.09	2.47 (0.26–23.6)	0.432
Wound complications	6 (5.9)	10 (7.1)	1.23 (0.43–3.50)	0.697	0.58 (0.17–2.06)	0.400
Inotropic use	48 (47.1)	89 (63.6)	1.96 (1.17–3.30)	0.011	1.18 (0.65–2.16)	0.582
Inotropic use, days	1.4 (±2.0)	3.4 (±5.4)				<0.001
Intensive care duration, days	1.8 (±1.1)	3.9 (±5.8)				<0.001
In-hospital duration, days	9.0 (±10.2)	15.4 (±22.7)				0.008

Values are n (%).

Outcomes* are analyzed by Cox regression analysis and HRs are reported.

Adjusted variables included age, male, ejection fraction, creatinine, hemoglobin, and off-pump technique.

UA; unstable angina; NSTEMI; non–ST-segment–elevation myocardial infarction; HR: hazard ratio; OR: Odds ratio; AKI: acute kidney injury; MACCE: major cardiovascular cerebral event (death, myocardial infarction, stroke, repeat revascularization).

The risk of postoperative AKI was significantly higher in the NSTEMI group (6.9% vs. 23.6%; OR, 2.76; 95% CI, 1.09–6.99; *p*-value = 0.032). Postoperative inotropic use was more frequently needed in the NSTEMI group based on univariate analysis, but it was not significant after adjustment. The durations of inotropic use, intensive care, and in-hospital stay were also longer in the NSTEMI group (1.4 days vs. 3.4 days, *p*-value < 0.001, 1.8 days vs. 3.9 days, *p*-value < 0.001, and 9.0 days vs. 15.4 days, *p*-value = 0.008, respectively). Rates of other postoperative complications such as newly-developed atrial fibrillation, reoperation, or wound complications did not significantly differ between the two groups.

**Figure 3:**
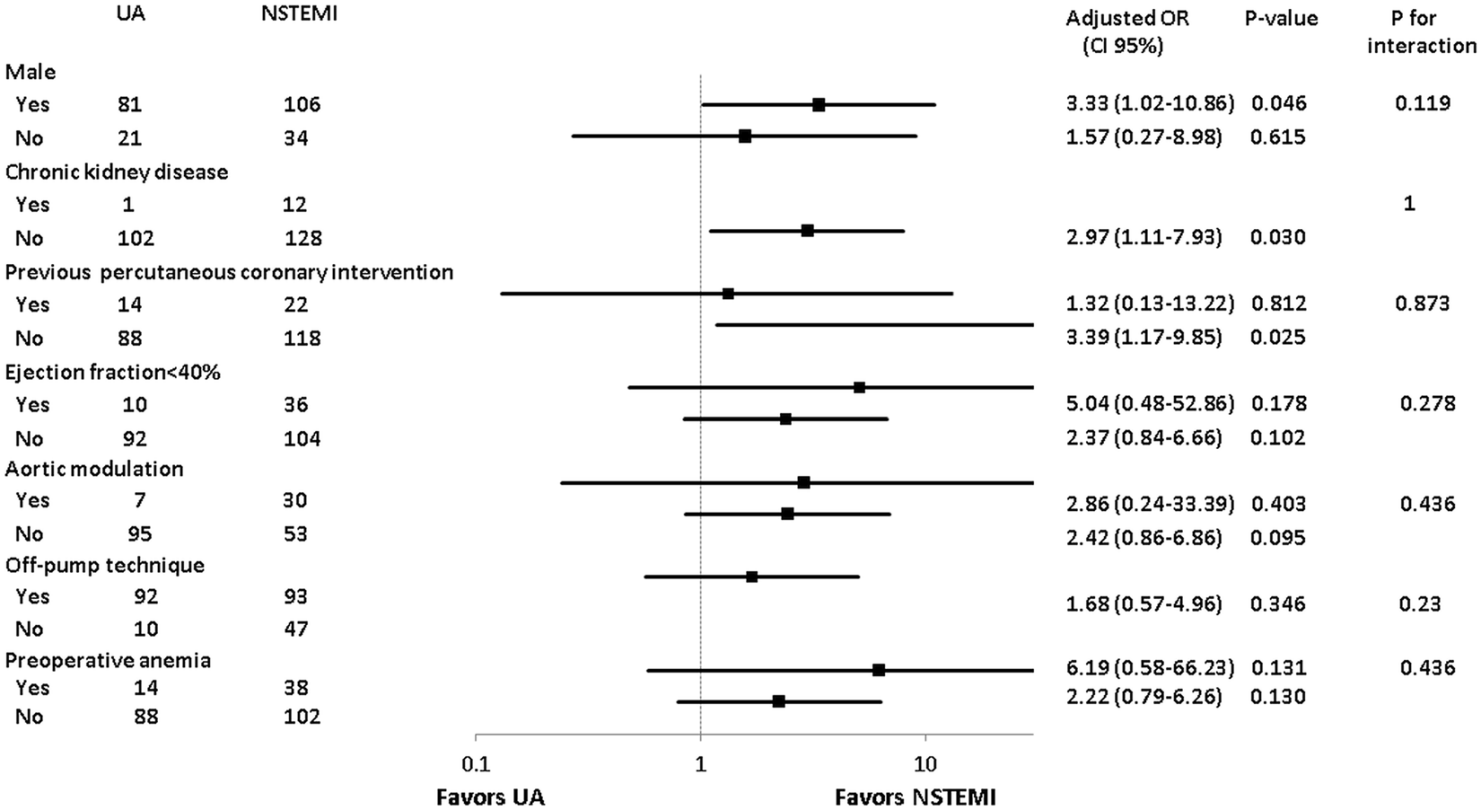
Subgroup analysis of male, chronic kidney disease, previous PCI, ejection fraction <40%, aortic modulation, and off-pump technique for postoperative acute kidney injury.

In subgroup analysis, there were no significant interactions between the presence of MI and variables of interest such as being male, chronic kidney disease, previous PCI, ejection fraction <40%, aortic modulation, off-pump technique, and preoperative anemia with respect to postoperative AKI. The results of subgroup analyses are shown in forest plots (Fig. 3).

## Discussion

The main findings of the present study are as follows: 1) compared to the UA group, the NSTEMI group showed a higher incidence of postoperative AKI and longer durations of inotropic use, intensive care, and hospital stay after eCABG, 2) the incidence of MACCE and other complications were not significantly different between the groups. These results suggest that, in NSTE-ACS patients, hs-cTnI measured at the ED may be associated with the postoperative complications of eCABG, but the incidence of complications during long-term follow-up might not be different.

By the beginning of the 21^st^ century, UA was included as a subgroup of ACS, along with NSTEMI and STEMI. UA and NSTEMI exhibit the same clinical features and have been usually considered together as NSTE-ACS, but differentiating the subgroups of NSTE-ACS has shown clinical importance in many previous studies^12,13^. Advances in the sensitivity of cardiac troponin assays resulted in reduction of URL, and led to high sensitivity but low specificity for detecting MI^14^. A currently available assay, with the URL reduced to 0.04 ng/ml, has not only shown clinical significance, but has also been validated in the ED setting^3,4,15^. Combined with clinical assessment, serial hs-cTnI testing was initially used for early exclusion of ACS in the ED^16,17^, and adopted in the accelerated diagnostic protocol for ED triage with satisfying results among patients with possible ACS^18,19^. However, guidelines for surgical coronary revascularization still lack detail.

In patients with STEMI, eCABG is strictly reserved for those with ongoing ischemia after successful or failed PCI, coronary anatomy suitable for CABG, mechanical complications of MI, or hemodynamic instability^6^. On the other hand, CABG in NSTE-ACS is justified in patients with extended ischemia, acute presentation of symptoms, and the ability to achieve full revascularization. Subgroups that require eCABG represent challenging and high-risk characteristics^8^. Modalities of revascularization in NSTE-ACS have never been directly compared in a randomized trial, but the recent American and European guidelines both suggest that, when hemodynamically stable, the modality can be chosen as if the patient had stable ischemic heart disease^5–7^. However, current guidelines do not provide an optimal timing of CABG for NSTEMI. Previous studies report that CABG is performed in 5 to 10% of patients with NSTE-ACS, and especially in NSTEMI patients one-third undergo CABG within 48 hours after hospital admission^7,8,20^. Although it is recommended to delay CABG for NSTEMI, recent studies report that outcomes of eCABG offer comparable results to those of staged CABG after adjusting for higher risk factors that are more likely to be shown in the patients who require eCABG^21^. So, up to the present it is reasonable to individualize the timing of CABG based on various factors^8^, and understanding the clinical impact of hs-cTnI at the ED on eCABG might be helpful. In this study, we used a current-generation assay, and performed serial testing combined with clinical assessments in accordance with current protocols^18,19^ and showed that the clinical outcome of eCABG in NSTE-ACS was not different according to the presence of myocardial ischemia. Further studies on optimal timing of CABG or modalities for coronary revascularization in NSTE-ACS patients are needed.

The results of this study showed that the immediate complications in the NSTEMI group, in terms of AKI, were more frequent compared to patients with UA, and it resulted in prolonged duration of inotropic use, intensive care, and hospital stay. Postoperative AKI is known to be caused by hemodynamic, inflammatory, and nephrotoxic factors, and independently associated with clinical outcomes^22^. Our results partly correlate with previous studies showing that hs-cTnI in the ED predicts early discharge^18,19^. However, contrary to previous studies on long-term outcomes, AKI did not change the incidence of MACCE^23^. This may share an explanation with one showing reduced incidence of postoperative AKI by using the off-pump technique in CABG but without evidence of better preserved kidney function or improved clinical outcomes^24^. In our study, the off-pump technique was more frequently used in the UA group; however, it was adjusted in the multivariate model and showed no interaction in subgroup analysis. Another explanation may be the magnitude of AKI. A reduction in the inflammatory response to change long-term outcomes might have been done for too few patients in this study, because the most frequent postoperative AKI was stage 1. Also, the difference in postoperative inotropic requirements suggests that lowered cardiac output may have caused a transient creatinine elevation. Although sustainment of AKI can be differentiated by optimized postoperative care, most previous studies did not refer to it^23^. Also, NSTEMI group showed higher incidence of ongoing ischemic symptoms as an indication for eCABG. This is reasonable because prolonged ischemia leads to myocardial infarction even despite the absence of evidence for coronary obstruction defined as type 2 myocardial infarction^25^. This may also be related to not only AKI but also more inotropic use and longer duration intensive care and hospital stays in the NSTEMI group. However, the incidence of MACCE did not differ despite higher incidence of preoperative ongoing ischemic symptoms. So, our results suggest that eCABG could be performed in these patients with more caution and optimized postoperative care.

Although a significant rise or fall in serial testing of hs-cTnI maintains the high diagnostic accuracy of myocardial infarction^26^, renal dysfunction itself can also cause hs-cTnI elevation without a coronary cause^27^. In addition, despite the long-term survival benefit of early CABG, even in high-risk patients, cardiac troponin level alone cannot diagnose cardiac conditions^28^. So, a subgroup analysis was performed to evaluate hidden interactions with AKI risk factors, and revealed no interaction with any factor.

The results of this study should be interpreted considering the following limitations. First, this study was not randomized; therefore, potential confounding factors might have significantly biased the results. Second, although our practice followed institutional protocol based on current guidelines, details of surgical indications for eCABG may have not been always consistent. The main indication for emergency operation was summarized based on medical record, but measurable parameters for our indications are absent. And also, conditions other than pathology such as patient consent or the schedule of operating theaters may have also been involved. In addition, patients might have undergone different treatments in the ED, and details of treatment in the ED and postoperative care may have also been updated during the study period. Third, this study does not provide any evidence for supporting eCABG in NSTE-ACS patients, and is rather focused on the effect of myocardial ischemia on eCABG. Moreover, non-inferiority of our analysis showed low statistical power because of small study population. Therefore, a following investigation is needed to change details of daily practice. Despite these limitations, this is the first study to compare outcomes of eCABG among the subgroups of NSTE-ACS classified by hs-cTnI in the ED.

## Conclusion

In eCABG for the patients presenting ongoing pain of a potential cardiac ischemia without ST elevation, UA and NSTEMI patients showed comparable outcomes, but hs-cTnI elevation at the ED was associated with immediate postoperative complications. A further study is needed to investigate optimized postoperative care.

## Methods

### Study population and data collection

This study was a single-center study approved by the Institutional Review Board at Samsung Medical Center. From January 2012 to March 2016, 1,792 consecutive patients who underwent CABG at our institution were initially enrolled. Inclusion criteria were 1) patients who visited the ED for chest pain, 2) patients with hs-cTnI measured in the ED, and 3) patients who underwent eCABG. We excluded one patient younger than 18 years of age considering the different reference limit for hs-cTnI in pediatric patients. To exclude hs-cTnI elevation from direct manipulation of the heart, we also excluded 23 patients who underwent percutaneous coronary intervention (PCI) or cardiac massage from presentation at the ED to eCABG. Enrolled patients were grouped according to serum hs-cTnI level measured in the ED (UA [<0.04 ng/mL, n = 102] and NSTEMI [≥0.04 ng/mL, n = 140]). In patients with multiple measurements, the peak level was used for stratification. The need for individual consent was waived by the Institutional Review Board, as this was a retrospective study involving medical record review. All medical data were collected by a standardized protocol, and analyzed anonymously after deidentification.

### Preoperative hs-cTnI level

Hs-cTnI is routinely used at our institution, and is measured using a highly sensitive immunoassay by an automated analyzer (Advia Centaur XP, Siemens Healthcare Diagnostics, Erlangen, Germany). All patients who presented to the ED with possible ACS underwent serial testing of hs-cTnI according to our institutional protocol. The lower limit of detection was 0.006 ng/mL, and the upper reference limit (URL), according to the 99th percentile reference, was 0.04 ng/mL. Reference limits were all provided by the manufacturer.

### Surgical and anesthetic management

The patients for eCABG were selected at the discretion of a surgeon and cardiologist on duty based on the current guidelines. The major indications of eCABG included (1) ongoing chest pain, (2) coronary anatomy unsuitable for PCI, and (3) ongoing or recurrent ischemia. The ongoing chest pain was defined as intractable or worsening pain. Coronary anatomy was determined as unsuitable either by cardiologists based on coronary angiogram or when PCI was attempted but failed. Ischemia was regarded as ongoing when ischemic symptoms such as pain, dyspnea or shortness of breath were accompanied by cardiac enzyme elevation, EKG change, cardiac enzyme elevation, fatal arrhythmia, heart failure or vital instability. Other indications for eCABG were other pathologic condition of the patient or schedule of operating theater. The indications of eCABG are summarized in Supplemental Table 1. CABG procedures and postoperative management were standardized according to our institutional protocol. Briefly, CABG was performed through a median sternotomy. The off-pump technique was our primary choice, but in patients with severe hemodynamic instability during coronary anastomosis, an intra-aortic balloon pump and cardiopulmonary bypass were used. The internal thoracic artery, previously known as the mammary artery, was used as the primary graft, and other vessels such as the greater saphenous vein were used for coronary conduits if needed. Angiographic follow-up was performed on the first postoperative day to assess graft patency. All patients were reevaluated at 1, 3, 6, and 12 months after surgery.

Details of anesthetic techniques have been previously described elsewhere^29^. After cannulating the radial artery for real-time blood pressure monitoring during induction of general anesthesia, all patients were induced and maintained either with propofol and remifentanil or etomidate and isoflurane inhalation. The femoral artery and internal jugular vein were cannulated for direct hemodynamic monitoring. Mechanical ventilation using a mixture of medical air and oxygen was set at a tidal volume of 8–10 ml/kg, and the respiratory rate was adjusted to maintain normocapnea. Crystalloids and colloids were infused in response to hemodynamic changes.

### Definitions and outcomes

ECABG was defined as a procedure performed within 48 hours after presentation at the ED. The primary outcome was major adverse cardiovascular and cerebral events (MACCE), which was defined as a composite of all-cause mortality, myocardial infarction (MI), repeat revascularization, and stroke. MI was defined according to the third universal definition of clinically relevant MI^25^. Stroke was defined as a new ischemic or hemorrhagic lesion causing a neurological deficit lasting longer than 24 hours. The secondary outcomes included MACCE, postoperative acute kidney injury (AKI), newly-developed atrial fibrillation, reoperation, postoperative inotropic treatment, and wound complications during the hospital stay. The durations of inotropic use, intensive care, and hospital stay were also compared. Postoperative AKI was defined based on the Kidney Disease Improving Global Outcomes criteria using creatinine level. An absolute increase more than 0.3 mg/dl or a relative increase more than 50% from preoperative baseline level was definitive for AKI^30^.

Past medical history was organized by reviewing the electronic medical records. Hypertension was self-reported or prescription of anti-hypertensives or systolic blood pressure >140 mm Hg at rest. Diabetes mellitus was defined as a history of treatment such as medication and lifestyle intervention or diagnosis of type 1 or type 2 diabetes mellitus. The history of stroke was defined as a history of neurological function loss caused by an ischemic or hemorrhagic event with residual symptoms at least 24 h after onset. Chronic kidney disease was defined as any condition with gradual loss of kidney function with serum creatinine level consistently over 2.0 mg/dl or being on dialysis. Anemia was defined as a hemoglobin level less than 13 g/dL in men and less than 12 g/dL in women.

### Statistical analysis

For continuous variables, the Student’s t-test or Mann-Whitney test was used, and the results were presented as mean ± standard deviation (SD). Chi-square or Fisher’s exact test was used to compare categorical data. Kaplan-Meier estimates were used to generate survival curves which were compared with the log-rank test. Covariates with a *p*-value < 0.15 were used in the multivariable model. We performed multiple Cox (MACCE) or logistic regression (AKI, atrial fibrillation, reoperation, wound complication, and inotropic use) analysis and hazard ratios (HR) or odds ratios (OR) are reported with 95% confidence intervals (CI). For Cox regression analysis, the time-to-event period was calculated as months. The power of an equivalence test for the difference between the groups was calculated using the non-inferiority margin (p-value at non-inferiority margin of 5.01% = 0.02). To further adjust the difference between the two groups, we performed subgroup analysis to reveal hidden interactions between postoperative AKI and variables such as male, chronic kidney disease, previous PCI, ejection fraction <40%, aortic modulation, off-pump technique, preoperative anemia.

All statistical analyses were performed using SPSS 20.0 (SPSS Inc., Chicago, Illinois, USA). All tests were two-tailed and p-values < 0.05 were considered statistically significant.

## Supplementary information

Supplemental Table 1
