# Supplementary material for: Association between high-sensitivity cardiac troponin I measured at emergency department and complications of emergency coronary artery bypass grafting
Source: Sci Rep. 2019 Nov 15;9:16933. doi: 10.1038/s41598-019-53047-y (PMC6858436; doi:10.1038/s41598-019-53047-y)
Supplement: Supplementary file 1 — Supplemental Table 1 [file 41598_2019_53047_MOESM1_ESM.pdf]

Association between high-sensitivity cardiac troponin I measured at emergency department and complications of emergency coronary artery bypass grafting

Jungchan Park, MD[1], Seung-Hwa Lee, MD[2], Jeong Jin Min, MD[1], Jong-Hwan Lee, MD[1], Ji Hye Kwon, MD[1], Ja Eun Lee, MD[1], Jin-Ho Choi, MD[2,3], Young Tak Lee, MD[4], Wook Sung Kim, MD[4], Myungsoo Park, MD[5], Ji Su Jang, MD[6] & Sangmin Maria Lee, MD[1]

[1]Department of Anesthesiology and Pain Medicine, Samsung Medical Center, Sungkyunkwan University School of Medicine, Seoul, Korea

[2]Division of Cardiology, Department of Medicine, Heart Vascular Stroke Institute, Samsung Medical Center, Sungkyunkwan University School of Medicine, Seoul, Korea

[3]Department of Emergency Medicine, Samsung Medical Center, Sungkyunkwan University School of Medicine, Seoul, Korea

[4]Department of Thoracic and Cardiovascular Surgery, Samsung Medical Center, Sungkyunkwan University School of Medicine, Seoul, Korea

[5]Department of Internal Medicine, Hallym University Dongtan Sacred Heart Hospital, Hwaseong, Korea

[6]Department of Anesthesiology and Pain Medicine, College of Medicine, Kangwon National University, Gangwondaehak-gil, Chuncheon-si, Gangwon-do, Republic of Korea

J.Park and S.H.Lee contributed equally to this work.

Disclosures: None.

Correspondence: Sangmin Maria Lee, MD, Department of Anesthesiology and Pain Medicine, Samsung Medical Center, Sungkyunkwan University School of Medicine, 81 Irwon-ro, Gangnam-gu, 06351 Seoul, Korea.

Tel: +82-2-3410-0362; Fax: +82-2-3410-0361; E-mail address: maria3run@gmail.com

Additional information

Disclosure: The authors declare no conflicts of interest.

Funding: None

Supplemental Table 1. Indications of emergency coronary artery bypass grafting

	**UA (N = 102)**	**NSTEMI (N = 140)**
Ongoing chest pain	70 (68.6)	41 (29.3)
Unsuitable anatomy for coronary intervention	7 (6.9)	12 (8.6)
Ongoing or recurrent ischemia	22 (21.6)	87 (62.1)
Other	3 (2.9)	0

UA; unstable angina; NSTEMI; non–ST-segment–elevation myocardial infarction
